# Application of Concretes Made with Glass Powder Binder at High Replacement Rates

**DOI:** 10.3390/ma14143796

**Published:** 2021-07-07

**Authors:** M. Isabel Más-López, Eva M. García del Toro, Sara García-Salgado, Daniel Alcala-Gonzalez, Santiago Pindado

**Affiliations:** 1 Departamento de Ingeniería Civil: Construcción, Infraestructura y Transporte ETSI Civil, Universidad Politécnica de Madrid Alfonso XII, 3, 28014 Madrid, Spain; mariaisabel.mas@upm.es; 2 Departamento de Ingeniería Civil: Hidráulica y Ordenación del Territorio ETSI Civil, Universidad Politécnica de Madrid Alfonso XII, 3, 28014 Madrid, Spain; sara.garcia@upm.es (S.G.-S.); d.alcalag@upm.es (D.A.-G.); 3 Instituto Universitario de Microgravedad “Ignacio Da Riva”, ETSI Aeronáutica y del Espacio, Universidad Politécnica de Madrid, 28040 Madrid, Spain; santiago.pindado@upm.es

**Keywords:** sustainability, sustainable concrete, glass powder, deactivate concrete, leachates, exterior pavements

## Abstract

Glass is a material that can be reused, except for a small part that, due to its residual characteristics, cannot be reused and becomes a nonbiodegradable waste to accumulate in landfills. The chemical composition and pozzolanic properties of waste glass are encouraging for the use of these wastes in the cement and concrete industries and for providing technically and environmentally viable solutions. In this study, we propose the production of deactivated concretes with a high content of glass powder in the binder. The substitution percentage of glass powder for cement used in this work was between 70% and 80%. Consistency, air content, bulk density, workability, compression strength, and permeability tests were performed. Regarding compressive strength, the results obtained at 90 days for percentages of cement substitution by glass powder of 70 and 80%, respectively, were 14.2 and 8.6. The chemical analysis of leachates showed concentrations of Fe, Cu, V, Ni, and Mo, in mg L^−1^, of 1.57, 1.38, 0.85, 0.95, and 0.44, respectively. The results obtained, compared with the relevant legislation, have proved that the inclusion of glass powder in a high percentage of substitution and with a granulometry of 20 µm in the manufacture of deactivated concretes is feasible for exterior pavements.

## 1. Introduction

Currently, the industrial sector generates large quantities of waste, part of which is recycled and the other part of which is deposited in landfills, causing environmental impacts [1]. These wastes have been the subject of numerous studies in recent years to determine possible uses based on their composition. In this way, the aim is, on the one hand, to reduce the effects on the environment by using these wastes and converting them into raw materials for other processes, thus reducing the exploitation of natural resources, and on the other hand, to generate new products in a cheaper way [2].

The sustainability of the civil engineering sector is crucial to drive society toward a circular economy, and to make this possible, the application of the waste hierarchy principle (prevention, preparation for reuse, recycling, recovery, and, as a last option, disposal) [3] is a priority. This sector, which advances day by day, is in a constant search for the best alternatives to provide solutions to the different market requirements [4]. The aim is for structures to be as resistant as possible and to ensure a certain useful life and optimum performance of the materials used without losing sight of the environmental aspect [5].

Concrete is by far the most widely used material in civil engineering. It is estimated that around 10 billion tons of this material are produced worldwide each year, which involves the use of nonrenewable natural resources, a significant demand for energy, as well as the emission of greenhouse gases [6]. For example, the production of one ton of Portland cement releases approximately one ton of carbon dioxide (CO_2_) into the atmosphere. Globally, the cement industry contributes 7% of the CO_2_ generated [7].

The traditional use of concrete is to build load-bearing structures due to its mechanical properties, durability, and workability. In recent times, the general commitment to sustainability has prompted a search for new concretes that can improve their properties in terms of environmental protection and aesthetic performance.

Numerous studies have shown the good performance of mortars and concretes made from different waste materials, especially waste glass [8,9,10].

The final glass wastes from packaging, demolition of buildings, the automotive industry, sanitary containers, and the ceramic industry that are not reusable by the glass industry can be recovered as raw material for the manufacture, after a process of fine grinding and mixing with nonpolluting reagents, of hydraulic binders [11].

Despite all benefits indicated, in the use of glass powder as a binder in the manufacture of concrete, it should be considered that these materials might contain potentially harmful substances such as heavy metals and trace elements, which can contaminate surface water that are often sources of drinking water supply [12,13,14]. In this sense, studies carried out show that the concentrations of heavy metals contained in the leachates emitted by different types of concrete do not endanger the quality of the aquifers [14,15,16].

Studies on the pozzolanic activity of waste glass carried out by Shao et al. [17] showed that glass powder ground to a particle size lower than 38 mm had some pozzolanic activity. Concrete made with 30% glass powder as a binder showed lower compressive strength before 28 days, but higher strength at 90 days [18]. This change in setting behavior of mortars and concretes made with glass powder composite binder, compared to mortars and concretes made with conventional binders (Portland cement), is attributed to the pozzolanic reaction of the glass powder [19]. It is shown that the pozzolanic activity of glass powder increases with decreasing particle size of the glass powder and increasing curing temperature [20,21]. The dissolution of the alkalis provided by the glass particles causes the cement hydration processes to be accelerated based on the amount of glass powder used in the manufacture of cement [22,23]. However, the amount of alkalis released is insufficient to compensate for hydration and early strength reduction caused by cement dilution [24].

Hongjian et al. [9] studied the properties of cements manufactured with a high percentage of glass powder replacement (above 60%) and concluded that all mixtures containing cement manufactured with a replacement percentage above 30% showed pozzolanic reactions after one year. This translates into a longer setting time and a self-healing capacity against the appearance of small cracks or differential settling that conventional cements do not have [24]. Más et al. [25] indicated the suitability of the use of concretes manufactured with a percentage of glass powder higher than 50% for pavements.

The range of pavements includes deactivated concrete, exposed aggregate, and washed concrete. This is a type of paving that is easy to install on site. The aggregates are visible on the surface in a concrete screed. This type of pavement, in addition to its mechanical function, can have an infinite number of finishes. This makes possible to obtain greater slip resistance and to improve the aesthetic component of concrete, until now considered by many as something gray and smooth [26]. Deactivated concrete slabs manufactured with a high percentage of glass dust in the binder, represent an ecofriendly alternative and in line with the circular economy, which is currently imposed as a principle in civil engineering.

In this article, we propose the use of glass powder as a high percentage cement substitute in the manufacture of deactivated concrete to be used as pavement in outdoor areas, studying its mechanical characteristics and leaching to verify that it does not cause a negative environmental impact on soils.

## 2. Materials and Methods

### 2.1. Materials

#### 2.1.1. Aggregates

**Figure 1:**
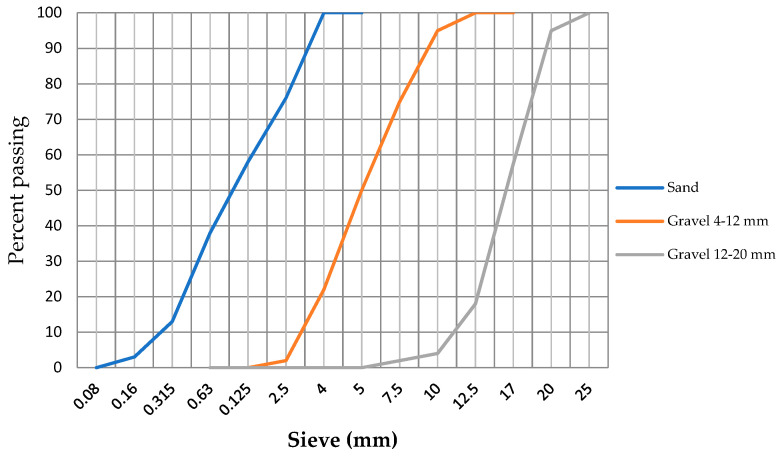
Aggregate granulometry.

Aggregates, essentially siliceous and nonreactive, are used. They are sand with a grain size <4 mm, gravel 4–12 mm, and gravel 12–20 mm. Figure 1 shows the particle size curve of these aggregates.

#### 2.1.2. Cement

The cement used was a commercial Portland cement CEM I 52.5 R (Cementos Portland Valderrivas, Morata de Tajuña, Madrid, Spain). This cement has a density of 3.12 g/cm^3^, a specific surface area of 4440 cm^2^/g, and a greenish gray color. The particle size of cement CEM I 52.5 R in volume fraction for particle diameter lower than 8 µm was 41.5% and for particle diameter lower than 96 µm was 99.7%.

Chemical composition of CEM I 52.5 R cement was as follows: CaO (65%), SiO_2_ (19%), Al_2_O_3_ (5.5%), FeO_3_ (2.65%), SO_3_ (2%), MgO (2%), Na_2_O (0.15%), K_2_O (0.7%).

Glass powder

The material used is the last fraction of glass that cannot be reused by the glass industry. This residue is ground with a bar mill until a grain size of 21 µm is obtained. The size was based on the results of the analysis in the COULTER LS 100 Q laser particle sizer (Beckman Coulter, Inc., Brea, CA, USA). Table 1 shows the diameters of the glass powder used in making concrete.

**Figure 2:**
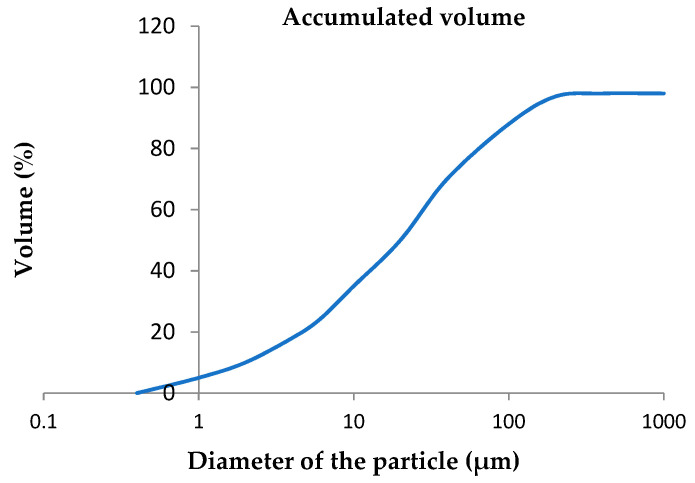
Distribution of the accumulated granulometry of glass powder.

Based on the results of the analysis in the particle sizer, the accumulated particle size curve was obtained, which shows the different particle size distribution as can be observed in Figure 2.

The chemical composition of glass powder was 72.00% SiO_2_, 11.85% Na_2_O, 11.19% CaO, 2.38% Al_2_O_3_, 1.60% Fe_2_O_3_, 1.60% MgO, 0.60% K_2_O, 0.10% MnO, 0.08% TiO_2_, and 0.05% P_2_O_5_, with 0.90% volatile LF (loss of the fire).

### 2.2. Sample Preparation

Two series of specimens, each of which had 15 units [27], were manufactured where the only parameter that varies is the replacement rate in percentage of glass powder by cement CEM I 52.5 R. Table 1 shows the formulation used in the manufacture of the specimens, where G70 and G80 correspond, respectively, to substitution rates of 70% and 80% of glass powder for cement. d_50_ glass powder of 21 μm was used (dimension of sample particles for which 50% of them have a diameter lower than a certain value). Table 2 shows a summary of experimental conditions for the samples prepared.

To study the compressive strengths of the manufactured concretes, they were introduced into 10 × 30 cylindrical test tubes. Once compacted and after 24 h, they were removed from the mold and placed in a humid curing chamber at a temperature of 20 °C. The curing time ranged between 28 and 90 days. After these curing times, they were broken following the UNE 83-304-84 standard [28] and their compressive strengths were measured.

### 2.3. Characterization Tests

1. Consistency test

It was carried out according to the UNE-EN12350-2 [29] consistency standard by means of the settlement test. This slump test is sensitive when the average slump is between 10 and 200 mm.

2. Air content

The air content of ready-mixed concrete was determined according to UNE-EN 12350-7 [30] by pressure methods.

3. Apparent density

The procedure followed for the calculation of densities and porosities is based on UNE-EN 1015-6 [31].

4. Workability

Workability was measured using the procedure described in UNE-EN 12350-5 [32].

5. Permeability test

Permeability tests were performed according to UNE-EN 12390-8 [33]. The purpose of this test was to evaluate the presence of chemical elements in leachate from filtration water and to study their possible environmental effects. Measurements were carried out on concrete preserved in endogenous medium at 20 °C for 100 h. The control was a cylinder of 4.07 ± 0.01 cm in length and 3.92 ± 0.01 cm in diameter. The water injection pressure was kept constant at 10 bar throughout the test.

In order to analyze the leached elements, a vacuum filtration was conducted. The filtrations were collected in successive fractions of ±20 cm^3^ through a 0.7 μm glass filter. They were later analyzed using atomic absorption spectrometry (FAAS) and inductively coupled plasma mass spectrometry (ICP-MS).

### 2.4. Slab Manufacturing

With the formulations shown in Table 1 (G70 and G80), 10 decorative concrete slabs were produced in 50 cm × 50 cm removable wooden molds. Five slabs were manufactured with the G70 formulation and the other five with the G80 formulation. The manufacturing was carried out by vibrocompacting using a vibrating tray with uniform pressure on the mold.

In order to make the aggregate visible on the surface of the plates, two methods were used. The first one consisted of deactivating the surface setting process by applying a deactivating product during the hours after the concrete has been placed. This chemical product was then removed by water jetting to expose the aggregate surface grains. The final appearance depends on the type and the amount of deactivator used, and the time of application before rinsing. In this case, a surface deactivator used for concrete floors with exposed aggregates similar to the one indicated by the Pieri VBA 2002 trademark (Figure 2). The application time for our tests was 2 days.

**Figure 3:**
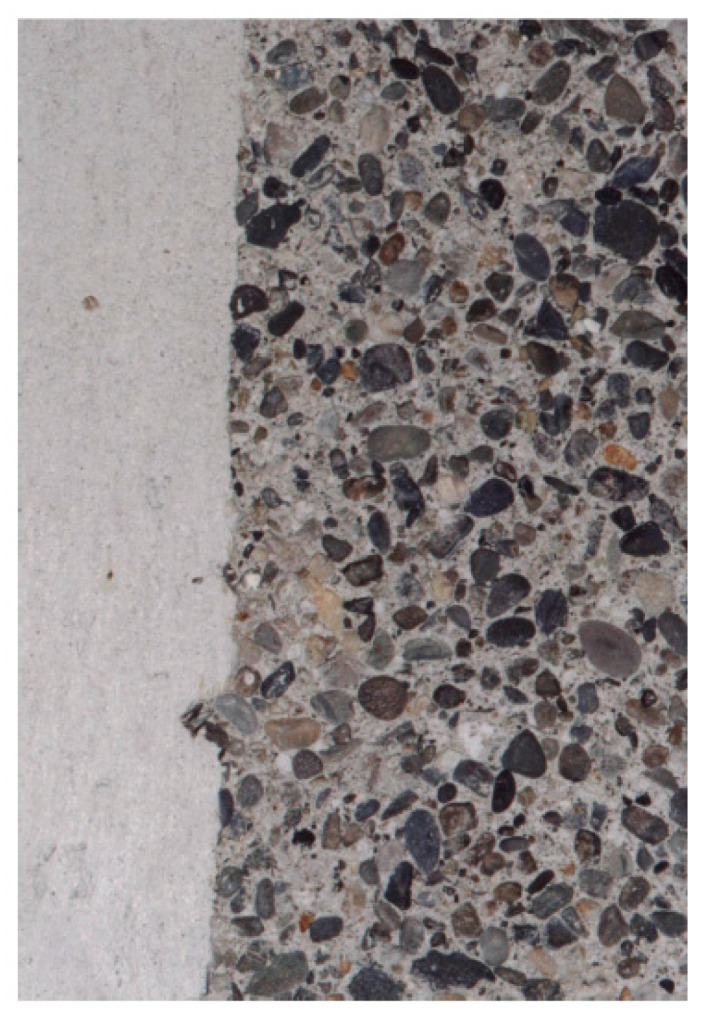
View of a deactivated decorative concrete slab with a binder composed of 80% glass powder and 20% CEM I 52.5 R cement. The left side of this photograph shows the appearance of the concrete before applying the chemical or mechanical treatment.

**Figure 4:**
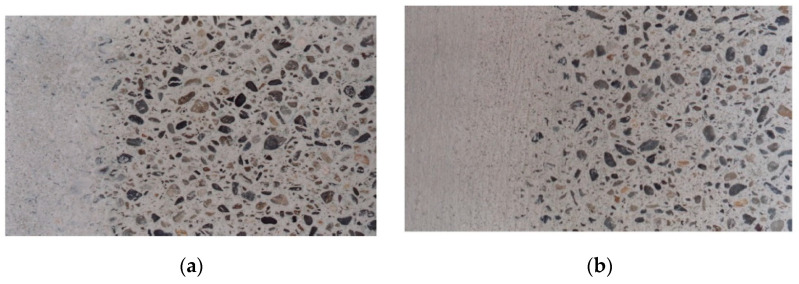
(**a**) View of a swept decorative concrete slab with a binder composed of 70% glass powder and 30% CEM I 52 R cement. (**b**) View of a swept decorative concrete slab with a binder composed of 80% glass powder and 20% CEM I 52 R cement. The left side of each one of these photographs shows the appearance of the concrete before applying chemical or mechanical treatments.

The second one was a mechanical method consisting of passing a broom over the concrete surface during the hours following its placement, in order to drag a thin layer of mortar or grout, thus revealing the aggregate grains on the surface. This method allowed the work to be finished immediately after sweeping. The final appearance varied according to the intensity of the sweeping (Figure 3 and Figure 4). The broom could be used as soon as the concrete surface was sufficiently dry. This surface sweeping was possible during the first hours after the laying of the concrete thanks to the slow setting time due to the presence of the glass powder in the binder.

## 3. Results and Discussion

### 3.1. Results of the Characterization of Concrete

The percentage of glass powder substitution by cement was based on the study carried out by García del Toro et al. [34], who reported that concretes manufactured with a percentage of cement substitution by glass powder higher than 50% were suitable to be used as pavements. The percentages of 70% and 80% were chosen in order to reuse a greater amount of waste and contribute in a greater extent to the circular economy and environmental protection.

Results obtained for consistency, air content, apparent density, and workability tests are shown in Table 3.

From Table 3, it can be observed that the air content and consistency are the same for concretes made with glass powder regardless of the percentage of substitution, and in both cases, they are lower than for the control specimen, in which all the binder is cement. As for the bulk density, it can be stated that it decreases when the proportion of cement substitution by glass powder in the binder increases. This is explained by the fact that the density of cement is higher than that of glass powder, 3.12 g/cm^3^ compared to 2.54 g/cm^3^ for glass powder. As for workability, it can be observed that as the percentage of glass powder in the mix increases, the concrete becomes more workable, a fact that is also related to the lower density of glass powder compared to cement.

According to consistency tests (Abrams cone slump), these concretes can be qualified as suitable for pavements. It can also be observed that the water content of these concretes is somewhat lower than that of conventional concretes in order to obtain a drier concrete. The air content is higher due to the presence of ground glass. The presence of air is convenient for this product since it facilitates its workability. On the other hand, it causes a decrease in compressive strength in the short term.

### 3.2. Mechanical Properties of Concrete

The results of the compressive strength tests of the concrete with 70% and 80% substitution of cement by glass powder are shown in Table 4.

As previously observed [34], for concrete prepared with cement replaced by glass powder, the compressive strength of the concrete decreased as the amount of glass powder in the binder increased, which can be attributed to the fact that glass powder has a low pozzolanic activity at the early ages, although, in this case, the granulometry is larger and the concretes are drier and with higher air content. This causes the appearance of different C-S-H type hydrates and slower setting [31] and lower compressive strengths in the short term.

### 3.3. Results of Permeability Tests

In a previous work [16], a preliminary study was carried out on permeability characteristics in this kind of concretes. In the present work, this test was further performed, obtaining similar results. In the first hours of the test, there was a rapid increase in the flow rate. This was due to the incomplete saturation of the control before the start of the test and to the absorption of water by the binder, a process that gradually decreased. After 2 h 30 m, the filtrate flow rate decreased before stabilizing and then increased slightly. This decrease in the filtrate flow rate corresponded to the internal reorganization of the free solid particles inside the core, some of which were blocked in the narrowing of the pores. This reorganization and the pushing of the finest particles caused a slight increase in the flow rate and, consequently, in the permeability of the core. This indicated that the C-S-H type gels responsible for the setting of the mortar began to form.

Regarding leaching characteristics, the elements analyzed in the leachate samples were as follows: Si, Na, K, Al, Ca, Li, B, Mg, Sc, Ti, V, Cr, Mn, Fe, Co, Ni, Cu, Zn, Ga, Ge, As, Rb, Sr, Y, Zr, Nb, Mo, Pd, Ag, Cd, Sn, Sb, Cs, Ba, La, Ce, Pr, Nd, Sm, Yb, Lu, Hf, Ta, Tl, Pb, Th, and U.

The results below are the average of the results obtained by the two detection methods used: atomic absorption spectrometry (FAAS) and inductively coupled plasma mass spectrometry (ICP-MS).

**Figure 5:**
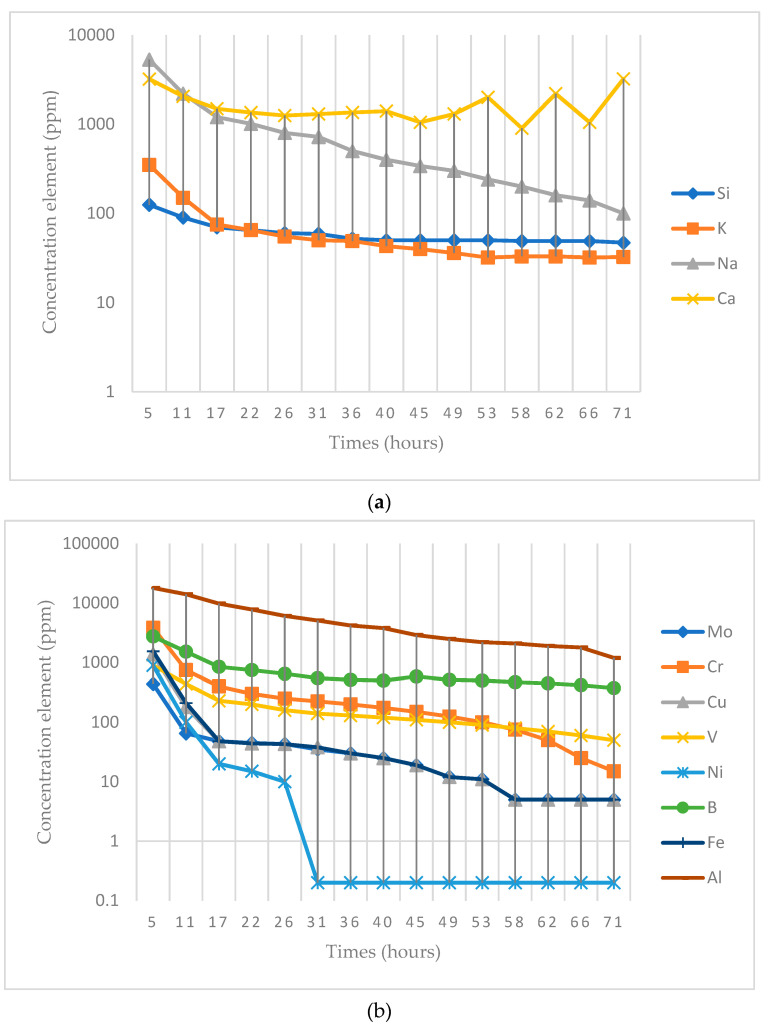
Evolution of the concentration (logarithmic scale) of the elements with the highest concentrations present in the mortar filtration water. (**a**) Concentrations of Si, K, Na, and Ca; (**b**) concentrations of Mo, Cr, Cu, V, Ni, B, Fe, and Al.

Most of the elements showed no relevant concentrations in the first hours of the test, reaching not detectable concentrations at the end of the test. For the rest of elements, in order to study their concentration in the leachates and their variation over time, they were divided into two groups. We took potentially contaminating elements (Figure 5b), which can be dangerous for soils and aquifers, and the rest of the elements found (Figure 5a) into account. The graphical representation of experimental data in these figures has been carried out in a logarithmic scale for a better visualization of data.

The potentially hazardous elements—molybdenum, chromium, copper, vanadium, nickel, iron, boron, and aluminum—were all found in the glass composition. The elements iron, copper, vanadium, nickel, and molybdenum showed concentrations of 1.57, 1.38, 0.85, 0.95, and 0.44 ppm, respectively, after five hours of the test, with a decreasing tendency until they stabilized after 20 h. At 72 h, their concentrations remained below the quantification limits of the analytical techniques used.

Figure 5b shows how the boron concentration was appreciable at the beginning of the test, decreasing rapidly with time from 2.75 to 0.37 ppm. Chromium was also detected in the first hours, with a concentration of 3.85 ppm. This concentration decreased rapidly to a concentration of 0.015 ppm at the end of the test. Aluminum was another component of the glass used in the test, at a percentage of 2.2%, so it also appeared in the filtered water. The concentration of this element, as can be seen in Figure 5b, decreased rapidly from 17.5 ppm to 1.15 ppm. The cement, consisting of 5.5% aluminum, also contributed a certain amount.

For the rest of the elements, and in order to verify that the results obtained in this work were in agreement with those obtained in a preliminary study by Mas et al. [16], a hypothesis test for equality of means with a confidence level of 95% was performed to ensure that there were no significant differences between the results. First, the variances of each mean were compared using the *F* test and it was found that there were no significant differences between them. At this regard, the hypothesis statement was as follows:

(1) $$H_{0}:{\bar{X}}_{1}={\bar{X}}_{1}$$

(2) $$H_{1}:\;{\bar{X}}_{1}\neq {\bar{X}}_{1}$$

(3) $$\mathrm{The}\;\mathrm{criteria}\;\mathrm{to}\;\mathrm{accept}\;H_{0}\;\mathrm{is}:\;|t_{0}|\;<\;t\;\mathrm{tabulated}\;$$

As previously mentioned, the hypothesis contrast was performed for the elements whose concentrations at 72 h remained above the quantification limits of the analytical techniques used.

Table 5 shows the results obtained in the previous test performed by Mas et al. and the current ones, together with the experimental Student’s *t* used to test the hypothesis.

Since the Student’s *t* test result obtained with the experimental data for each and every one of the elements analyzed was lower than the tabulated data for a Student’s *t* distribution with ν degrees of freedom and a 95% confidence level, it was accepted that there were no significant differences for the means of the data obtained for each of the elements analyzed.

Of all the elements, sodium (Na) was the one released in the greatest quantity; its concentration in the filtered water was around 5350 ppm in the first 5 h of the test. Figure 5a shows how the sodium concentration decreased rapidly to around 100 ppm at the end of the test. This result was obtained for sodium mainly because this element is found in a high percentage in the glass used (≈11.3%), and also because sodium has high mobility. However, the cement used contains only 0.15% sodium, contributing little to the concentration. As for potassium, Figure 5a shows how the concentration of this element dropped from 359 ppm to 32 ppm. This element came from both glass and cement, whose potassium content is 0.6% and 0.7%, respectively. As for silicon, which is also shown in Figure 5a, it can be observed that its concentration decreased more slowly than the rest of the elements, from 112 to 47 ppm. The silicon collected in the filtrate water came from both glass (70%) and cement (19%). The calcium concentration, as shown in Figure 5a, varied differently over time. At the beginning of the trial, there was a decrease in calcium concentration from 31 to 1.2 ppm. Subsequently, the concentration remained constant until 30 h have elapsed. After this time, the concentration increased until it reached a final value of 3.2 ppm. According to Marco et al. [11] the dissolution of the glass did not release calcium, so it can be concluded that the calcium collected in the filtration came to a large extent from the cement, since cement is made up of 65% Ca.

Sodium and potassium, especially sodium, mainly from glass, were released in significant quantities, but this does not pose a danger to the environment, since they were not released in sufficient quantity to cause changes in the electrical conductivity of the soil. Calcium does not constitute an environmental hazard; since it is a nontoxic element and at the concentration level determined, it would not cause soil pH modifications. The presence of chromium and boron during the first hours of the test in the filtrate water was relevant. However, these elements tended to disappear at the end of the test, probably because they got into the cementitious matrix.

## 4. Conclusions

In the present work, the use of glass powder as a high percentage cement substitute in the manufacture of deactivated concrete to be used as pavement in outdoor areas was examined. Deactivated concrete slabs manufactured with a high percentage of glass dust in the binder represent an ecofriendly alternative that is in line with the circular economy, which is currently imposed as a principle in civil engineering.

The experimental results indicated that it is feasible to produce mortar with fine glass powder and that floors made of deactivated concrete with a glass powder binder base are highly stable against atmospheric and mechanical agents. These characteristics give them a long-life cycle and low maintenance. Exposed aggregate finishes are rough, nonslip, and resistant to wear and tear and the action of atmospheric agents. Its application would be suitable for pedestrian areas such as park streets and outdoor traffic areas in need of a durable pavement, as well as accesses to garages, terraces and patios, swimming pool areas, and roads with light traffic.

Regarding leachates, alkaline elements, especially sodium, were released in significant quantities, although not in quantities sufficient to cause changes in the electrical conductivity of the soil, so they do not pose an environmental hazard, similar to calcium, which is a nontoxic element and, at the concentration level determined, would not cause a soil pH modification. The considerable presence of chromium and boron, potentially contaminating elements, in the filtrate water during the first hours of the test is remarkable. These elements tended to disappear at the end of the process, since they were incorporated into the cement matrix; therefore, they do not cause environmental contamination. Finally, the rest of the elements detected were found at trace levels in the filtered water, so due to their scarcity, they cannot constitute a danger to the natural environment according to the legislation in force.

## Figures and Tables

**Table 1:** Granulometric characteristics of glass powder.

Glass Powder Used		
d_10_	d_50_	d_90_
20 ± 0.01 µm	21 ± 1 µm	76 ± 3 µm

**Table 2:** Summary of experimental conditions for the samples prepared.

	Sample ID		
Concrete Composition	G70	G80	Control
Cement substitution rate for glass powder (%)	70	80	0
Cement CEM I 52,5 R (kg/m^3^)	99	66	330
Glass powder (kg/m^3^)	231	264	0
Equivalent binder (kg/m^3^)	330		
w/c ratio (%)	0.52		
Arid < 4 mm	740		
Gravel 4–12 mm	310		
Gravel 12–20 mm	850		

**Table 3:** Results obtained in the characterization of fresh concrete and concrete substituted by glass powder.

	G70	G80	Control
Consistency (mm)	0	0	11
Air content (%)	7.1	7.1	2.8
Apparent density (kg/m^3^)	2098	2051	2390
Workability (easy/difficult)	Difficult	Easy enough	Easy

**Table 4:** Average results of compressive strength, Cs, measured in concrete specimens in relation of the time after setting.

Days	Compressive Strength (MPa)		Control
	G70	G80	0
7	5.6	1.5	29.9
28	10.0	3.7	34.5
90	14.2	8.6	39.6

**Table 5:** Quantitative results for analyzed elements, expressed as mg L^−1^ (mean ± standard deviation, *n* = 3) and Student’s *t* values obtained for the contrast of means.

Element	Experimental Value (ppm)	Results Obtained by Mas et al. [16] (ppm)	$S^{2}=\frac{\left(n_{1}-1\right)S_{1}^{2}+\left(n_{2}-1\right)S_{2}^{2}}{n_{1}+n_{2}-2}$	$t_{0}=\frac{{\bar{X}}_{1}-{\bar{X}}_{2}}{\sqrt{S^{2}\left(\frac{1}{n_{1}}+\frac{1}{n_{2}}\right)}}$
Na	97 ± 3	100 ± 3	9.46	1.35
Si	45 ± 3	47 ± 4	13.11	0.65
K	31 ± 2	32 ± 2	3.71	0.70
Ca	3.2 ± 0.3	3.2 ± 0.5	0.17	0.15
Al	1.36 ± 0.09	1.2 ± 0.2	0.02	1.28
B	0.30 ±0.02	0.38 ± 0.05	0.002	1.63

Student *t* values were 2.1318 for all the elements.

## Data Availability

Not applicable.

## References

[1] Mohajerani A., Vajna J., Cheung T.H.H., Kurmus H., Arulrajah A., Horpibulsuk S. (2017). Practical recycling applications of crushed waste glass in construction materials: A review. Constr. Build. Mater..

[2] David A., Thangavel Y.D., Sankriti R. (2019). Recover, recycle and reuse: An efficient way to reduce the waste. Int. J. Mech. Prod. Eng. Res. Dev..

[3] Ginga C.P., Ongpeng J.M.C., Daly M.K.M. (2020). Circular economy on construction and demolition waste: A literature review on material recovery and production. Materials.

[4] Yu W., Cheng S.T., Ho W.C., Chang Y.H. (2018). Measuring the sustainability of construction projects throughout their lifecycle: A Taiwan Lesson. Sustainability.

[5] Chau K.W. (2007). Incorporation of sustainability concepts into a civil engineering curriculum. J. Prof. Issues Eng. Educ. Pract..

[6] Pade C., Guimaraes M. (2007). The CO_2_ uptake of concrete in a 100 year perspective. Cem. Concr. Res..

[7] Hendrik G., van Amy C. (2008). Padovani Cement Manufacture and the Environment Part II: Environmental Challenges and OpportunitiesNo Title. J. Ind. Ecol..

[8] Jani Y., Hogland W. (2014). Waste glass in the production of cement and concrete—A review. J. Environ. Chem. Eng..

[9] Du H., Tan K.H. (2017). Properties of high volume glass powder concrete. Cem. Concr. Compos..

[10] Ahmad S., Xu A. (2007). Performance of glass powder as a pozzolanic material in concrete: A field trial on concrete slabs. Cem. Concr. Res..

[11] Marco J., Garcia E., Más M.I., Alcaraz V., Luizaga A. (2012). Estudio de la resistencia a compresión de morteros fabricados con conglomerante compuesto de polvo de vidrio. Inf. Constr..

[12] Hohberg I., de Groot G.J., van der Veen A.M.H., Wassing W. (1997). Development of a leaching protocol for concrete. Stud. Environ. Sci..

[13] Głuchowski A., Sas W., Dziecioł J., Soból E., Szymaňski A. (2018). Permeability and leaching properties of recycled concrete aggregate as an emerging material in civil engineering. Appl. Sci..

[14] Diotti A., Galvin A.P., Piccinali A., Plizzari G., Sorlini S. (2020). Chemical and leaching behavior of construction and demolition wastes and recycled aggregates. Sustainability.

[15] Marion A.M., De Lanève M., De Grauw A. (2005). Study of the leaching behaviour of paving concretes: Quantification of heavy metal content in leachates issued from tank test using demineralized water. Cem. Concr. Res..

[16] Mas M.I., García E.M., Marco L.J., De Marco J. (2016). Análisis de la Viabilidad Ambiental de la Utilización de Morteros Fabricados con Polvo de Vidrio en la Estabilización de Suelos. Inf. Tecnol..

[17] Shaoa Y., Lefort T., Morasa S. (2000). Damian Rodriguez Studies on concrete containing ground waste glass. Cem. Concr. Res..

[18] Lu J.X., Duan Z.J., Poon C.S. (2017). Combined use of waste glass powder and cullet in architectural mortar. Cem. Concr. Compos..

[19] Taha B., Nounu G. (2008). Properties of concrete contains mixed colour waste recycled glass as sand and cement replacement. Constr. Build. Mater..

[20] Mirzahosseini M., Riding K.A. (2014). Effect of curing temperature and glass type on the pozzolanic reactivity of glass powder. Cem. Concr. Res..

[21] Maraghechi H., Maraghechi M., Rajabipour F., Pantano C.G. (2014). Pozzolanic reactivity of recycled glass powder at elevated temperatures: Reaction stoichiometry, reaction products and effect of alkali activation. Cem. Concr. Compos..

[22] Strength C. (2001). Chemical Reactions of Glass Cullet as cement component. J. Mater..

[23] Rashidian-Dezfouli H., Afshinnia K., Rangaraju P.R. (2018). Efficiency of Ground Glass Fiber as a cementitious material, in mitigation of alkali-silica reaction of glass aggregates in mortars and concrete. J. Build. Eng..

[24] Islam G.M.S., Rahman M.H., Kazi N. (2017). Waste glass powder as partial replacement of cement for sustainable concrete practice. Int. J. Sustain. Built Environ..

[25] Más-López M.I., del Toro E.M.G., Patiño A.L., García L.J.M. (2020). Eco-friendly pavements manufactured with glass waste: Physical and mechanical characterization and its applicability in soil stabilization. Materials.

[26] Lasalle E., Escuela Técnica Superior de Ingeniería de Caminos Canales y Puertos (2008). Hormigones desactivados: Hormigones con árido visto en pavimentos y hormigones decorativos en prefabricados industriales. Jornadas Sobre hormigones Especiales.

[27] AENOR UNE-EN 123501-1 (2006). Ensayos de Hormigón Fresco.

[28] AENOR (1985). Ensayos de Hormigón;Rotura por Comprensión: Norma Española: UNE 83-304-84.

[29] UNE 12350-2 (2006). Ensayos de Hormigón Fresco. Ensayo de Asentamiento.

[30] (2020). Comité Técnico CEN/TC 104 Hormigón y productos Relacionados UNE-EN 12350-7: Ensayos de hormigón fresco. Parte 7: Contenido de Aire Métodos de Presión.

[31] (1999). CTN 83—HORMIGÓN UNE-EN 1015-6:1999: Densidad Aparente del Hormigón Fresco.

[32] ANEFHOP UNE-EN 12350-5:2020 (2020). Ensayos de Hormigón Fresco. Parte 5: Ensayo de la Mesa de Sacudidas.

[33] UNE-EN (2020). Norma Española Parte 8: Profundidad de Penetración de Agua Bajo Presión.

[34] Del Toro E.G., Alcala-Gonzalez D., Más-Lópe M.I., García-Salgado S., Pindado S. (2021). Use of ecofriendly glass powder concrete in construction of wind farms. Appl. Sci..

